# Risk Assessment and Deployment for Safety Showers and Eyewash Stations in the Process Plant Industry

**DOI:** 10.3390/ijerph18168707

**Published:** 2021-08-18

**Authors:** Jae-Young Choi, Sang-Hoon Byeon

**Affiliations:** Department of Health and Safety Convergence Science, Korea University, Anam-ro 145, Seongbuk-gu, Seoul 02841, Korea; jaeyoungchoi@korea.ac.kr

**Keywords:** safety shower and eyewash station, ANSI Z358.1, process safety, operator safety, risk assessment

## Abstract

Safety showers and eyewash stations are equipment used for primary washing if their operator is exposed to hazardous chemicals. Therefore, safety showers and eyewash stations should be installed to ensure operator safety in process plants with excessive hazardous chemicals. International guidelines related to safety showers and eyewash stations are introduced in ANSI Z358.1, BS EN 15154, and German DIN 12899-3:2009, but only mechanical specifications regarding safety showers and eyewash stations are suggested. As such, there are currently no engineering guidelines, books, or technical journal papers requiring safety showers or eyewash stations and their efficient deployment. Thus, this study conducted risk assessment from an industrial hygiene perspective, suggesting which process equipment requires a safety shower and eyewash, including their economical and efficient deployment for operator safety. In industry, safety showers and eyewash stations are considered part of the process safety field; this study attempted to contribute to the safety improvement of operators by applying risk assessment of the industrial hygiene field. More studies are needed that contribute to operators’ safety by incorporating industrial hygiene fields for other process safety fields, including safety showers and eyewash stations.

## 1. Introduction

In process plants, safety showers and eyewash stations are installed as the primary protection if an operator is exposed to hazardous chemicals [1]. International guidelines for safety showers and eyewash stations include ANSI Z358.1, BS EN 15154, and German DIN 12899-3:2009. However, these guidelines only offer mechanical specifications for safety showers and eyewash stations and do not provide any information on which process equipment is protected by safety showers and eyewash stations. The International Safety Equipment Association also states that safety showers and eyewash stations should be deployed through risk assessment but do not provide detailed guidelines on how this should be implemented [2].

Therefore, this study suggests a guideline determining the type of process equipment that is protected by a safety shower and eyewash station and an efficient methodology for deploying it for economic efficiency and safety.

## 2. Materials and Methods

For the definition of process equipment requiring a safety shower and eyewash station to precede, safety showers and eyewash stations should be deployed considering economic efficiency and safety aspects. Installing safety showers and eyewash stations requires the cost of facility itself, the cost of material and construction to set up utility water pipe for operation of this facility, and the cost of the system to increase the capacity of utility water. Additionally, there may be electrical heat tracing costs to prevent freezing of utility water in cold areas such as Russia and chiller system costs to cool utility water in hot areas such as Saudi Arabia. In conclusion, because of the cost impact, it is not economical to install safety showers and eyewash stations indefinitely for the safety of the operator. Therefore, this study suggests dividing the two parts, the definition of process equipment requiring a safety shower and eyewash station and the actual placement of safety showers and eyewash stations. The reason for this approach is to install safety showers and eyewash stations only where it is needed to satisfy both efficiency and economic aspects.

### 2.1. Risk Assessment for Safety Shower and Eyewash Station Need

The risk assessment determining the need for safety showers and eyewash stations is based on the theoretical equation of industrial hygiene (Equation (1)) [3].

[Risk] = [Hazard] × [Exposure](1)

#### 2.1.1. Hazard Identification for Risk Assessment

In Equation (1), hazard means inherent property of an agent or situation having the potential to cause adverse effects when an organism, system, or (sub)population is exposed to that agent [4]. Additionally, exposure means concentration or amount of a particular agent that reaches a target organism, system, or (sub)population in a specific frequency for a defined duration [4]. Methods to identify the health risk of each chemical include using the health category according to National Fire Protection Association (NFPA) 704 and the “Globally Harmonized System of Classification and Labeling of Chemicals” (GHS) code on Material Safety Data Sheet (MSDS). The health category by NFPA 704 indicates that the chemical is classified into five categories based on the macroscopic perspective on health effects and the human body (Table 1) [5].

However, this type of classification is not suitable for defining safety showers and eyewash station targeting process equipment. This is because when an operator is exposed to hazardous chemicals, only materials with NFPA 704 health category 3 require safety showers and eyewash stations. As the operator could die upon very short exposure to NFPA 704 health category 4 materials, there is not enough time to clean them for first aid. So, there is no need to install safety showers and eyewash stations for process equipment handling NFPA 704 health category 4 materials. Therefore, for a detailed risk assessment, the health category of hazardous chemicals should include more details. This study determines the health hazards of each hazardous chemical through the GHS code [6].

The GHS follows the United Nations’ guidelines for the skin corrosion/irritation and eye effects categories. For skin corrosion/irritation, it is further classified into three subcategories, as shown in Table 2.

Eye effects are also classified into two subcategories, as shown in Table 3.

For the hazard aspect, which is required data to perform risk assessment for safety shower and eyewash station, it is a more accurate approach based on GHS rather than NFPA 497. However, there has been no previous engineering guidelines, books, or technical journal papers how to use this. Therefore, this study should consider how a hazard would be considered applying the actual operation of the process plant.

#### 2.1.2. Identification of Exposure for Risk Assessment

Although process plants are composed of various process equipment devices, specific types of process equipment have high operator maintenance rates (Table 4) [7].

Many engineering references specify that the process hazard analysis (PHA) technique in process plants provides an exposure category concept.

In this study, exposure was classified into five categories, referring to the guidelines of Saudi Aramco (Dhahran, Saudi Arabia) [8] and Abu Dhabi National Oil Company (ADNOC) (Abu Dhabi, United Arab Emirates) [9]; details are listed in Table 5.

The exposure category in Table 5 is based on data from Saudi Aramco and ADNOC, who have a long experience in the operation of the process plant. So, it is regarded as an international guideline in the process plant industry. Therefore, this exposure approach in risk assessment for safety showers and eyewash stations would be reasonable.

This study aims to suggest a methodology for how to perform risk assessment by combining modified hazard and exposure.

## 3. Result and Discussion

In this section, risk assessment methodology for safety showers and eyewash stations is suggested by applying and combining the data of hazard and exposure. For understanding, an example are introduced in each methodology. The advantage and limitations of the suggested methodology are also discussed.

### 3.1. Suggested Methodology for Risk Assessment of Safety Shower and Eyewash Station

Application of GHS for hazard grade would be suggested, and a combination of this hazard grade and exposure category also would be suggested by using a risk matrix approach. In addition, the concept of ALARP would be applied, and the methodology for efficient placement of safety showers and eyewash stations would be suggested, for example.

#### 3.1.1. Hazard Grade for Risk Assessment Based on GHS

As the eye is more sensitive to damage than the skin, the hazard graph was marked with a weight on eye irritability in this study, as shown in Table 6. The hazard was calculated by dividing it into five stages.

Chemicals, which do not have a separate category for skin corrosion/irritation and eye effects, were not considered in this study because they are not a material that requires a safety shower or eyewash station.

#### 3.1.2. Combination of Hazard and Exposure for Risk Assessment

In this section, a combination of hazard and exposure is suggested to decide what process equipment requires a safety shower and eyewash station. The risk graph methodology is used for the combination of hazard and exposure. This methodology is widely used in PHA techniques, such as a hazard and operability (HAZOP) study [10,11]. In this study, the concept of a hazard integrity level (HIL) was introduced as an indicator of the final grade and divided into four categories (Table 7).

When grading the HIL, the weight is placed on the exposure. Safety showers and eyewash stations are only used in the event of an accident; in normal operation conditions, the situation in which the operator is exposed to hazardous chemicals rarely occurs. Therefore, the exposure is weighted to complete the risk graph from a safety perspective.

For example, consider a process that uses trichloroethylene (TCE, CAS No. 79-01-6) where the equipment has a filter that must be manually replaced periodically. TCE has skin irritation category 2 and eye irritation category 2A (Table 2 and Table 3). So, the TCE hazard grade is 3 (see Table 6). The exposure category for this filter replacement is assumed to be 4 according to Table 5. Finally, using the matrix in Table 7, filter replacement of TCE handing equipment has an HIL of 3. Of course, if standard operating procedure (SOP) is applied in the filter replacement operation, little exposure of trichloroethylene may occur to the operator. However, from the perspective of process safety, exposure category 4 is applied by acknowledging the possibility of exposing the operator to trichloroethylene during the filter replacement operation.

#### 3.1.3. Applying the “as Low as Reasonably Possible” (ALARP) Concept for Risk Assessment

The ALARP concept is used to determine whether to apply actual improvements based on the final risk derived through various PHA techniques [12]. The application of safety equipment to all hazardous process equipment could be beneficial for safety, but it may not be realistic due to the financial costs.

Therefore, it is common for process plant owners to provide a standard where risks can be taken to a certain risk level according to the project size and financial margin. In this study, HIL 1 is considered the ALARP area, and the process equipment for requiring a safety shower and eyewash station is selected above HIL 2.

#### 3.1.4. Efficient Placement of Safety Shower and Eyewash Station

In the international ANSI Z358.1 guideline, safety showers and eyewash stations are to be installed within a walking distance of 10 s from when the operator is exposed to hazardous chemicals [13]. The walking distance of 10 s is considered to be 55 feet; global plant owners such as Saudi Aramco consider a point of 15 m to be a conservative distance [13,14]. In this study, 15 m is regarded as the walking distance to reach the safety shower and eyewash station from a safety perspective. If there is only one process equipment that requires a safety shower and eyewash station inside a 15 m coverage, there is no need to consider its efficient placement because the safety shower and eyewash station should be located as close as possible. However, if two or three process equipment areas are covered with a single safety shower and eyewash station inside a 15 m coverage, their efficient placement becomes significant.

In this study, if two or three process equipment is covered with a single safety shower and eyewash station, it should be positioned relative to the reciprocal of the HIL rating of each process equipment. For process equipment with a higher HIL rating, the operator should be able to reach the safety shower and eyewash station faster than with process equipment with a lower HIL rating.

Suppose two process equipment devices require a safety shower and eyewash station and that they are inside the 15 m coverage. Additionally, the HIL ratings of the two are 2 and 4, respectively. Under industry practice, the location of the safety shower and eyewash does not matter when the process equipment is inside the 15 m coverage of the safety shower and eyewash station. However, this approach does not provide a solution to how close the safety shower and eyewash should be to each piece of process equipment, despite the different exposure risks to the two different process equipment operators.

**Figure 1:**
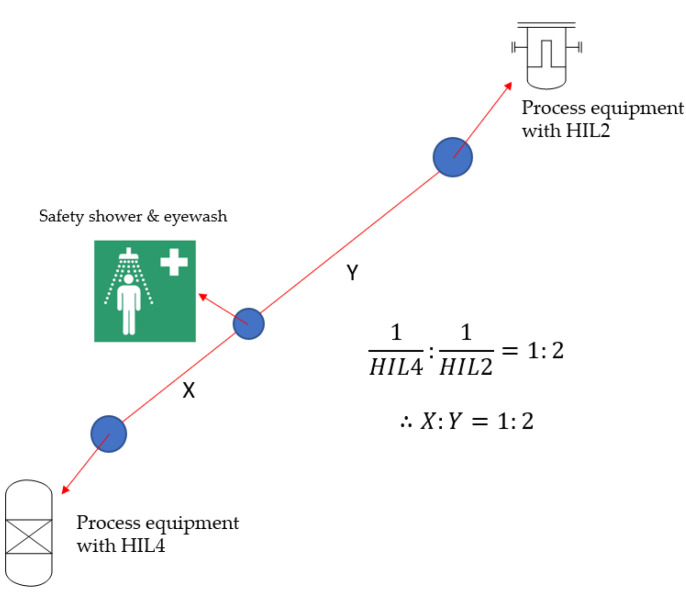
Example for efficient placement of safety shower and eyewash station.

However, when approached according to this study, a safety shower and eyewash station can be deployed considering the operator’s safety. The distance from the safety shower and eyewash to the process equipment of HIL2 and HIL4 ratings should be proportional to the reciprocal of the HIL rating; that is, the distance from the safety shower and eyewash to the HIL4 process equipment should be arranged such that it is half the distance from the safety shower and eyewash to the HIL2 process equipment. This arrangement allows the safety shower and eyewash station to be placed closer to the process equipment with a higher exposure risk to the operator, allowing for the efficient deployment of the safety shower and eyewash station (Figure 1).

**Figure 2:**
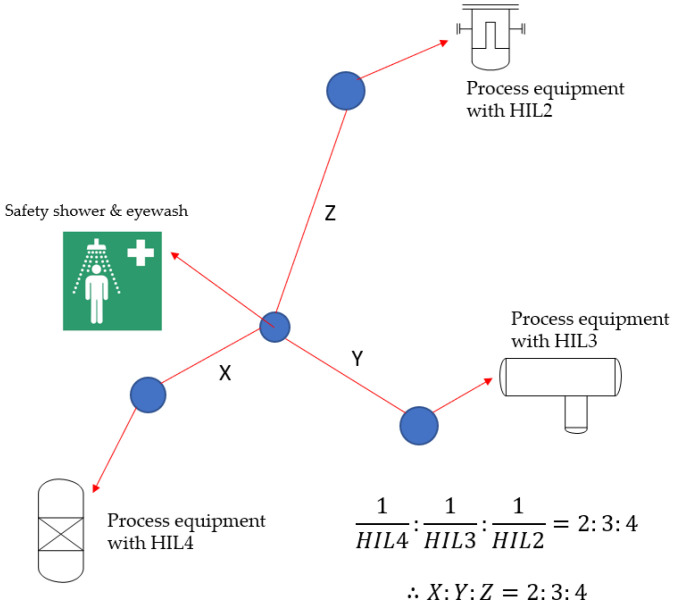
Application for other example of efficient placement of safety shower and eyewash.

In addition, even if three process equipment devices with different HIL ratings can be covered by one safety shower and eyewash, placing it according to Figure 2 can be an efficient arrangement considering the operator’s safety. Several applications are possible in the same manner.

### 3.2. Advantage and Limitation of Suggested Methodology

Since there is no technical journal paper for risk assessment of safety showers and eyewash stations, this study would be advantageous with the certain topic such as safety equipment. It could be applied as an example in risk assessment based on the other special topics in the process safety part.

However, the operation experience of the process plant would be a key factor in assessing the exposure category during the risk assessment process. Therefore, for more detailed risk assessment, there would be a limitation in assessing an exposure category only for applying brainstorming of the experienced process engineer and the operator, such as a HAZOP workshop.

### 3.3. Conclusions

In a process plant with hazardous chemicals, safety showers and eyewash stations must be installed to ensure operators’ safety. However, although risk assessment techniques for process plants are widely supported by engineering guidelines, books, and technical journal papers, risk assessments for safety showers and eyewash stations remain limited.

Thus, this study introduced a risk assessment technique from an industrial hygiene perspective, suggesting a methodology for installing safety showers and eyewash stations accurately and efficiently. This study presents a method for deployment or placement of safety showers and eyewash stations that prioritizes the riskiest exposures. This study also suggests the deployment of safety showers and eyewash stations to reduce operators’ exposure risk.

In the view of industrial hygiene, safety showers and eyewash stations have been treated as simply a kind of personal protective equipment, with little interest in detailed deployment. While industrial hygiene deals with the exposure risk of the operator working in the process plant, the deployment of safety showers and eyewash stations has been dealt with in the process safety field, so separate responsibilities may make safety improvements difficult to implement. However, this study incorporated an industrial hygiene risk assessment technique for the process safety field. Such a convergence between the industrial hygiene field and the process safety field should be attempted to improve operators’ safe working inside a process plant.

## Figures and Tables

**Table 1:** NFPA 704 health category.

Category	Description
0	Poses no health hazard, no precautions necessary, and would offer no hazard beyond that of ordinary combustible materials (e.g., wood, paper)
1	Exposure would cause irritation with only minor residual injury (e.g., acetone, sodium bromate, potassium chloride)
2	Intense or continued but not chronic exposure could cause temporary incapacitation or possible residual injury (e.g., diethyl ether, ammonium phosphate, carbon dioxide, iodine, chloroform, DEET)
3	Short exposure could cause serious temporary or moderate residual injury (e.g., liquid hydrogen, sulfuric acid, calcium hypochlorite, carbon monoxide, hexafluorosilicic acid, zinc chloride)
4	Very short exposure could cause death or major residual injury (e.g., hydrogen cyanide, phosgene, diborane, methyl isocyanate, hydrofluoric acid)

**Table 2:** Skin corrosion and irritation.

Skin Corrosion Category 1			Skin Irritation Category 2	Mild Skin Irritation Category 3
Destruction of dermal tissue: visible necrosis in at least one animal			Reversible adverse effects in dermal tissue Draize score: ≥2.3 < 4.0 or persistent inflammation	Reversible adverse effects in dermal tissue Draize score: ≥1.5 < 2.3
Subcategory 1A Exposure < 3 min Observation < 1 h	Subcategory 1B Exposure < 1 h Observation < 14 d	Subcategory 1C Exposure < 4 h Observation < 14 d		

**Table 3:** Eye effects.

Serious Eye Damage Category 1	Eye Irritation Category 2	
Irreversible damage 21 d after exposure. Draize score: Corneal opacity ≥ 3 Iritis > 1.5	Reversible adverse effects on cornea, iris, conjunctiva Draize score: Corneal opacity ≥ 1 Iritis ≥ 1 Redness ≥ 2 Chemosis ≥ 2	
	**Irritant** Subcategory 2A Reversible in 21 d	**Mild irritant** Subcategory 2B Reversible in 7 d

**Table 4:** Item list for manual operation.

Item Type	Description for Manual Handling
Chemical injection package	Chemical injection involves manual handling of the injection point connection and the connection to the chemical tank
Filter	Manual handling comprises the periodic replacement of filters
Reactor	If the reactor operates with a catalyst, the operator must perform manual handling by periodically replacing the catalyst
Tank loading/unloading	Injecting material from the tank or entering the tank involves a hose reel, which requires manual handling where workers may be exposed to the chemical

**Table 5:** Table for exposure category.

Category		Description
1	Very low	Never heard of in industry Unlikely to occur in the lifetime or multiple plants may occur in the lifetime of the industry
2	Low	Some incidents in the industry Expected to occur in the lifetime of multiple plants
3	Medium	Incident has occurred in the company Expected to occur once in the lifetime of a single plant
4	High	Happens several times per year in the company Expected to occur several times in the lifetime of the plant
5	Very high	Happens several times per year in the facility Expected to occur frequently in the lifetime of the plant

**Table 6:** Hazard graph for risk assessment.

		Skin Irritation				
		Category 1A	Category 1B	Category 1C	Category 2	Category 3
Eye irritation	Category 1	5	5	5	4	3
	Category 2A	5	5	4	3	2
	Category 2B	5	4	3	2	1

**Table 7:** Category table for hazard integrity level (HIL).

		Exposure				
		5	4	3	2	1
Hazard	5	HIL 4	HIL 4	HIL 4	HIL 3	HIL 3
	4	HIL 4	HIL 4	HIL 3	HIL 3	HIL 2
	3	HIL 4	HIL 3	HIL 3	HIL 2	HIL 2
	2	HIL 3	HIL 3	HIL 2	HIL 2	HIL 1
	1	HIL 3	HIL 2	HIL 2	HIL 1	HIL 1

## Data Availability

The data presented in this study are available on request from the corresponding author.
